# Variant pubertal development in Prader-Willi syndrome: early and slow progression of pubarche with normal age at gonadarche

**DOI:** 10.3389/fendo.2025.1527140

**Published:** 2025-04-15

**Authors:** Aneta Kodytková, Petra Dušátková, Shenali Anne Amaratunga, Stanislava Koloušková, Barbora Obermannová, Renata Pomahačová, Štěpánka Průhová, Marta Šnajderová, Zdeněk Šumník, Jiřina Zapletalová, Valerij Semjonov, Jan Lebl

**Affiliations:** ^1^ Department of Pediatrics, 2nd Faculty of Medicine, Charles University and Motol University Hospital, Prague, Czechia; ^2^ Department of Pediatrics, Faculty of Medicine, Charles University and University Hospital Pilsen, Pilsen, Czechia; ^3^ Department of Pediatrics, Faculty of Medicine, Palacky University and Olomouc University Hospital, Olomouc, Czechia; ^4^ Department of Statistics, Motol University Hospital, Prague, Czechia

**Keywords:** Prader-Willi syndrome puberty, pubarche, gonadarche, *MKRN3* gene, puberty, bone age

## Abstract

**Introduction:**

Prader-Willi syndrome (PWS) is primarily caused by a paternal microdeletion of the 15q11-q13 region, maternal uniparental disomy (mUPD) or unbalanced translocations. The *MKRN3* gene, located within 15q11-q13, is a master regulator of pubertal initiation. We aimed to compare variant pubertal onset and progression with recent normative data and to correlate it with abnormal *MKRN3* gene status.

**Methods:**

Age at pubarche, gonadarche, subsequent pubertal progression and bone age (BA) at gonadarche were investigated in 37 PWS patients (18 females) who already entered pubarche and/or gonadarche with median age 11.1 (95% CI: 6.4 – 18.8) years. All patients were re-tested to confirm genetic subtypes of PWS. The *MKRN3* gene was analyzed using single gene sequencing.

**Results:**

Out of 37 subjects, 22 had microdeletion and 15 mUPD. Regardless of genetic subtypes and *MKRN3* gene status, no correlation between genotypes and the pubertal pattern was found. They initiated pubarche early – girls at 7.4 (95%CI:6.4–8.4), and boys at 9.2 (8.2–10.2) years. The subsequent progression from PH2 to PH4 (pubic hair development) was prolonged to 3.7 years in girls (1.5–5.9;p<0.05), and 2.9 in boys (2.2–3.6;p<0.001). The age at gonadarche was adequate – 10.0 years in girls (8.8–11.2), and 11.0 in boys (9.8–12.1). Progression rate of breast development from B2 to B4 was 3.9 (0.2–7.5) years in girls and of testicular volume from 4 ml to 15ml was 3.8 (0.0–8.1) years in boys. The BA at gonadarche is advanced by 0.6 ± 1.1 years (p<0.001).

**Conclusions:**

Children with PWS, regardless of the genetic subtype and/or *MKRN3* status, had an early pubarche and normally timed gonadarche. Pubarche progression was slower. Advanced BA was significantly correlated with gonadarche.

## Introduction

The genetic background for Prader-Willi syndrome (PWS) is abnormal genomic imprinting during gametogenesis, which causes a loss of paternal gene expression on chromosome 15 at the molecular level, in the 15q11-q13 region. About 65 – 75% of patients with PWS have a *de novo* paternal deletion within this critical region. Based on three breakpoints (BP) in this area, three classes of deletions may be distinguished – common type 1 deletion (BP1-3), type 2 deletion (BP2-3) and a rare atypical microdeletion 15q11.2 (BP1-2) (1, 2). An additional 20 – 30% of PWS patients exhibit maternal uniparental disomy (mUPD) of chromosome 15, and the remaining 1 – 3% of cases have microdeletion or epimutation in the imprinting center (1, 3, 4). Very rarely, an unbalanced Robertsonian chromosomal translocation may occur (5).

PWS, with an estimated incidence of 1 in 10,000 to 20,000 newborns, is the most common cause of syndromic life-threatening obesity (6, 7). Eating disorder in PWS can be divided into 4 phases: 1. hypotonia, feeding difficulties, and failure to thrive (from birth to 15 months of age); 2. weight gain without significant change in appetite or caloric intake or with only a slight increase in interest in food (from 15 months to 5 years); 3. development of hyperphagia, typically accompanied by food-seeking and lack of satiety (from 5 to 13 years); some adults progress to phase 4, which is the loss of an insatiable appetite and the achievement of a feeling of satiety (8). Several reports have demonstrated oxytocin abnormalities in PWS patients, which may be causal for several symptoms - poor suckling response at birth, hyperphagia with food addiction, poor social skills and emotional dysregulation. The results of animal studies indicate that oxytocin therapy is one of the important therapeutic strategies for children with PWS (9, 10). Excessive eating behavior in PWS appears to result from hypothalamic dysfunction, as do other key clinical features such as short stature, testicular retention due to hypogonadotropic hypogonadism, and, in some patients, central hypothyroidism and/or central hypocorticism (4). Furthermore, due to hypothalamic syndrome, pubertal development may be incomplete, delayed or completely absent and this can also lead to complications in the reproductive sphere. On the contrary, signs of central precocious puberty (CPP) have been reported in a minority (approximately 3.5%) of children with PWS (11–13).

The candidate gene for abnormal pubertal development in PWS is the maternally imprinted *MKRN3* that is located in 15q11.2, within the PWS critical region (14). *MKRN3* contains a characteristic ring zinc finger motif and encodes a specific protein (macorin ring finger protein 3) (15), which is abundantly expressed in the hypothalamus and is involved in hormonal regulation of puberty by the inhibition of gonadotropin-releasing hormone (GnRH) secretion (16, 17). In murine models, increased expression of *MKRN3* in the hypothalamus was reported in the neonatal and juvenile period with a subsequent decrease before pubertal onset, which is suggestive of its role to inhibit activation of hypothalamo-pituitary-gonadal axis (18). The inhibitory role of the MKRN3 protein is further supported by a decrease in its serum level in both girls and boys before the onset of puberty (19, 20), whereas girls with idiopathic CPP have lower serum MKRN3 when compared to prepubertal peers of similar age (21). The loss-of-function pathogenic variants in the *MKRN3* gene are the most common genetic cause of CPP (22–24).

Real-life clinical experience shows aberrant pubertal development in Prader-Willi syndrome, but clear data on the timing of the pubertal milestones have not been clearly elucidated yet. Even more, identification of the *MKRN3* gene within the Prader-Willi syndrome critical region as the master regulator of pubertal development led to the need of testing the potential link between the *MKRN3* gene expression and/or sequence and individual pubertal development in PWS patients. In this study, we aimed to estimate pubertal milestones in 37 PWS patients (18 females) with median age 11.1 (95% CI: 6.4 – 18.8) years on the background of recent normative data, to correlate bone age (BA) and gonadarche onset and evaluate the link between variant pubarche and gonadarche and the *MKRN3* gene sequence or expression level in individual genetic subtypes of PWS.

## Materials and methods

### Patients

This is a cross-sectional study including all available relevant retrospective data such as the onset of pubarche and/or gonadarche and their further progression, bone age at the time of gonadarche. All patients with genetically proven PWS who were currently treated by growth hormone (GH) between 2020 and 2021 in the three largest centers for GH treatment in Czechia and who already have entered pubarche (PH2) and/or gonadarche (glandular breast B2 or testicular volume 4 ml) were included in the study. Finally, a cohort of 37 children, adolescents, and young adults with median age 11.1 (95% CI: 6.4 – 18.8) years were evaluated with genetically confirmed PWS. Detailed information about the patients at the time of examination can be found in **Table 1**. All children started growth hormone (GH) treatment early in life at median age of 0.9 years (95% CI: 0.3 – 4.0 years). Their long-term clinical data have been collected according to a standard protocol every 6 months within the country-wide registry of GH recipients - database REPAR (25). Pubarche (PH2) and gonadarche (glandular breast B2 or testicular volume 4 ml) was evaluated according to Tanner (26, 27). One girl from our cohort (1/3) reached menarche and adult sexual maturity at an adequate age, but menarche was hormonally induced.

**Table 1:** Demographic data of PWS cohort at current age.

37 PWS patients	Age Mean (95% CI)	Height Mean (95% CI)	SDS Height Mean (95% CI)	BMI Mean (95% CI)	SDS BMI Mean (95% CI)	Duration of GH treatment Mean (95% CI)
18 girls	11.2 (6.3; 18.4)	150.8 (134.1; 169.5)	0.2 (-0.7; 1.1)	19.7 (17.2; 22.8)	1.5 (0.2; 2,1)	8.5 (4.8; 11.6)
19 boys	10.9 (7.9; 16.1)	149.9 (127.6; 186.2)	0.5 (-0.7; 1.7)	23.2 (21.0; 26.4)	1.6 (-0.3; 2.2)	10.1 (5.9; 13.3)

BA at gonadarche, based on a radiograph of the left hand and wrist, was assessed in 16 patients using the TW3 method (28).

All tested individuals and their parents gave written informed consent for enrollment into the study including genetic testing and all data used were anonymized. This study was approved by the Ethics Committee at the 2nd Faculty of Medicine, Charles University in Prague (date of approval: 27.10.2021; EK-1240.4/21). The research was conducted ethically in accordance with the World Medical Association Declaration of Helsinki.

### Normative data

Calculations of height and BMI SDS were based on the 6th Czech National-wide Anthropological Survey of Children and Adolescents (29, 30). Normative data for pubarche and gonadarche were obtained from recent Danish studies (31, 32). According to these population-based studies, mean age of girls at entry into stage B2 is 9.9 ± 1.3 years (95% CI: 9.7 – 10.0) and into stage PH2 is 11.1 (95% CI: 11.0 – 11.2) years (31). In boys, puberty starts at age 11.7 (95% CI: 11.5 – 11.8) years (testicular size more than 3 ml) and stage PH2 is achieved at 12.4 (95% CI: 12.2 – 12.6) years (32, 33).

### Genetic re-testing

All patients had their diagnosis of PWS already confirmed from previous genetic testing in different labs. For the purpose of this study, we re-tested all study participants. After obtaining informed consent, the patient’s blood was sampled, and DNA was extracted. Larger structural changes (deletions, duplications) causing PWS, as well as a different level of methylation in the *MKRN3* gene from the reference, were detected by methylation-specific MLPA (Multiplex Ligation Probe-Dependent Amplification; MRC Holland, Amsterdam, Netherlands; ME028 according to manufacturer’s instructions). Patients were divided into 3 groups according to the discovered structural change: type 1 deletion (BP1-3), type 2 deletion (BP2-3) and mUPD. The Sanger direct sequencing method and subsequent analysis using Mutation Surveyor software (SoftGenetics, State College, USA) were used to detect DNA sequence changes (substitutions, deletions, insertions) in the *MKRN3* gene.

### Statistical evaluation of pubertal milestones

All ages at pubarche and gonadarche were converted to SDS (31, 32). Deviations of pubarche/gonadarche below or over ± 2 SDS were evaluated as precocious or delayed, respectively. A sample t-test was used to compare age at pubarche and age at gonadarche (B2/testes 4ml) with the healthy population. One sample t-test was also used to evaluate the duration of progression of breast development, testicular volume progression and pubarche development compared to normative data. We performed a paired t-test, separately for girls and boys, to examine whether the onset of puberty (B2/testes 4ml) occurs later than the PH2 pubic hair stage in our patients. A Pearson correlation test was done to evaluate the linear relationship between bone age and calendar age at gonadarche and one tailed paired t-test was used to test the hypothesis that bone age is greater than calendar age at gonadarche.

## Results

### Genetic subgroups

Based on the results of MS-MLPA, out of 37 patients tested, in 22 (59%) PWS was caused by 15q11-q13 microdeletion – type 1 deletion in 9, and type 2 deletion in 13 patients, respectively. The remaining 15 (41%) patients had mUPD. The Sanger sequencing did not detect any (likely) pathogenic point variant within the *MKRN3* gene.

Regardless the genetic subtype, dose, or sequence of *MKRN3* gene no correlation was found between individual genotypes and the abnormal pattern of pubarche or gonadarche in PWS.

### Pubarche and gonadarche

Mean age at pubarche differs significantly between patients with PWS and healthy children (**Table 2**). In comparison with normative data, pubarche occurred 3.7 years earlier in girls, and 3.2 years earlier in boys (p<0.001; **Table 2**). On the contrary, gonadarche started at adequate age in both boys and girls (**Table 3**) compared to normative values.

**Table 2:** Age of pubertal milestones – the comparison of patients with PWS and healthy population (28, 29).

Pubertal milestone	Girls with PWS	Healthy girls	p-value	Boys with PWS	Healthy boys	p-value
Age at pubarche Mean (95% CI)	7.4 (6.4; 8.4)	11.1 (11.0; 11.2)	p<0.001	9.2 (8.2; 10.2)	12.4 (12.2; 12.6)	p<0.001
Age at gonadarche Mean (95% CI)	10.0 (8.8; 11.2)	9.9 (9.7; 10.0)	n.s.	11.0 (9.8; 12.1)	11.7 (11.5; 11.8)	n.s.

n.s., not significant.

**Table 3:** Duration of pubarche progression, breast development and testicular volume progression – the comparison of patients with PWS patients and healthy population (28, 29).

Pubertal progression [years]	Girls with PWS	Healthy girls	p-value	Boys with PWS	Healthy boys	p-value
PH2 to PH4 Mean (95% CI)	3.7 (1.5; 5.9)	1.4 (1.1; 1.7)	p<0.05	2.9 (2.2; 3.6)	1.3 (0.8; 1.7)	p<0.001
B2 to B4 Mean (95% CI)	3.9 (0.2; 7.5)	2.4 (2.1; 2.7)	n.s.	–	–	–
4 ml to 15 ml Mean (95% CI)	–	–	–	3.8 (0.0; 8.1)	2.3 (2.0; 2.8)	n.s.

n.s., not significant.

The BA at gonadarche tended to be advanced by 0.6 ± 1.1 years (p<0.001), there was significant correlation between skeletal advancement and age of gonadarche (Pearson correlation coefficient 0.8; p<0.001).

**Figure 1:**
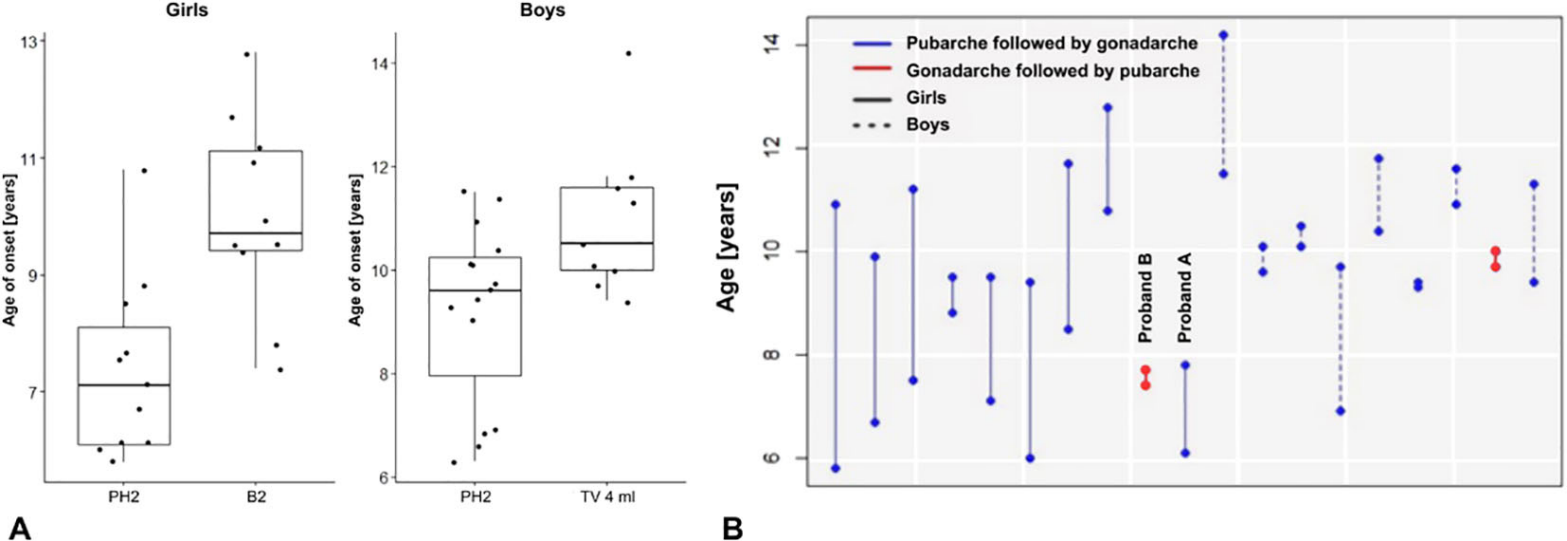
**(A)** Onset of pubarche and gonadarche in patients with PWS. **(B)** Differences in the age at onset of pubarche and puberty in 19 individuals (girls and boys) who already developed both - pubarche (PH2) and gonadarche (B2, testicular volume 4 ml). Only 2 girls had gonadarche followed by pubarche.

In patients with PWS, the pubic hair growth (stage PH2) started significantly earlier than the gonadarche (B2/testes 4ml) in both girls (p<0.001) and boys (p<0.01) – **Figure 1A**. The estimated mean difference between ages at pubarche and gonadarche was -2.5 in girls (95% CI: -3.6 to -1.4) and -1.2 in boys (95% CI: -2.0 to -0.4).

### Pubertal progression

**Figure 2:**
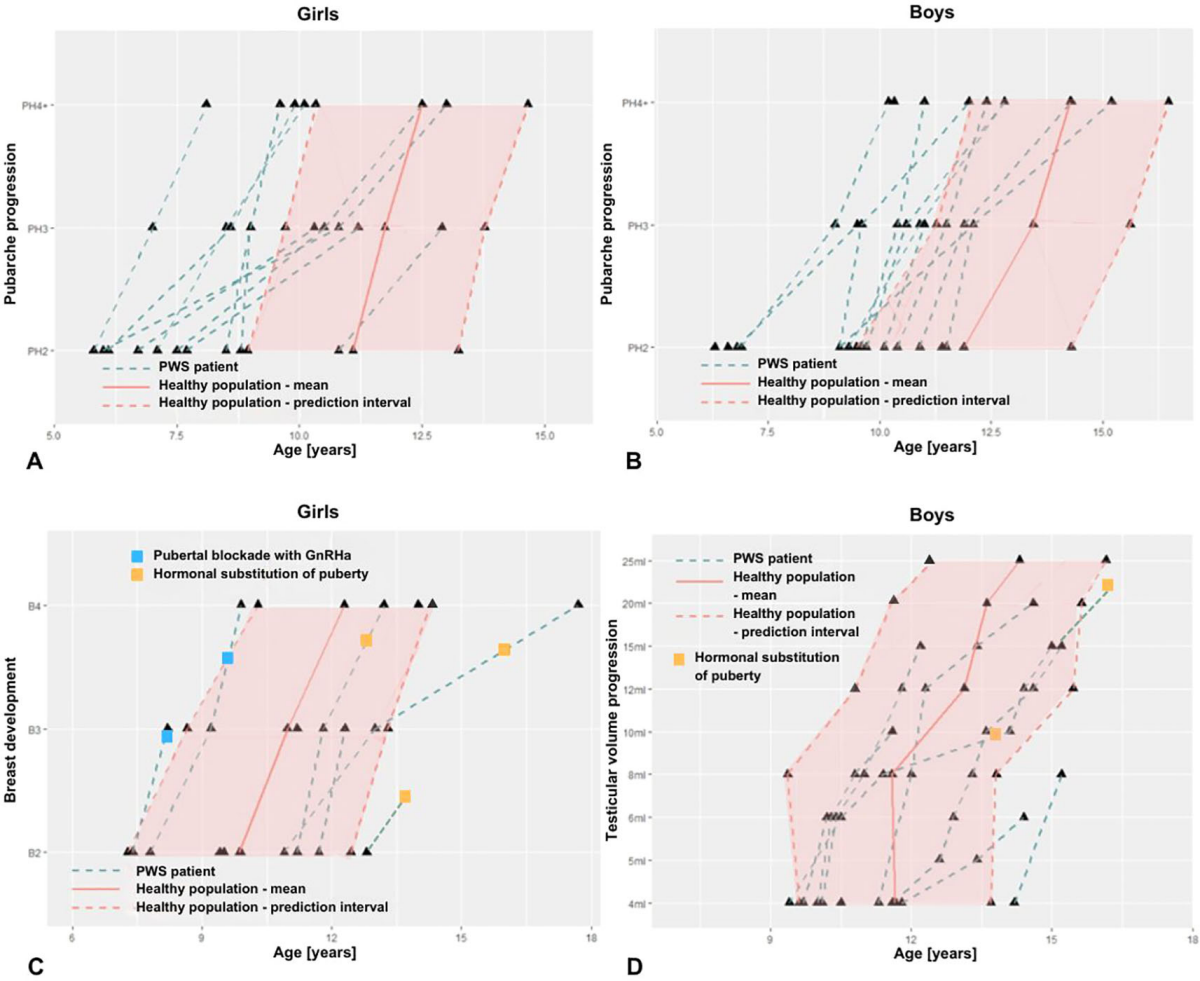
Onset and progression of pubic hair **(A, B)**, of breast maturation in girls **(C)** and of testicular volume in boys **(D)** with PWS compared to normative values (healthy population).

In patients with PWS, the average time of progression from PH2 to PH4 was significantly prolonged (**Table 3**), compared to the average time of pubarche progression in healthy girls and boys (**Figures 2A, B**), respectively.

Due to the limited number of patients within the late pubertal age, we failed to prove prolonged progression of breast development (stage B2 to B4), as well as, progression of testicular volume from 4 ml to 15 ml. In patients with PWS, there was no significant difference in duration of this period compared to the healthy population (**Table 3**, **Figures 2C, D**).

Hormonal replacement to accelerate non-advancing puberty was initiated in 4/5 adolescent girls at an average age of 13.7 years and in 2/3 adolescent boys at age 15.9 years (**Figures 2 C, D**). These children had spontaneous gonadarche at a physiological age, but further pubertal progression was insufficient, therefore hormone replacement was initiated at a later stage of pubertal development.

Two girls with mUPD developed rapidly progressing CPP with first clinical signs (stage B2) at ages 7.4 and 7.8 years. Both started receiving analogues of gonadotropin releasing hormone (GnRHa) by mean age 9.0 years. In one girl with CPP (proband A), puberty started with breast development followed by pubic hair consistently with the healthy population (**Figure 1B**). In the second girl with CPP (proband B) the pubertal pattern was typical for PWS – pubarche preceding gonadarche.

## Discussion

In this cross-sectional study combined with relevant retrospective data, we investigated 37 patients with PWS with spontaneous pubarche and/or gonadarche. We originally hypothesised that pathogenic variants in the *MKRN3* gene might be the cause of this abnormal pubertal pattern. *MKRN3* is included in the cluster of genes causative for PWS and is known to act as a repressor of puberty initiation (24, 34). If a loss-of-function variant in the *MKRN3* gene causes CPP, all patients with PWS caused by Type 1 and 2 deletions including *MKRN3*, would have to develop a CPP phenotype. However, we were unable to confirm this hypothesis, the percentage of those PWS adolescents with CPP is similar to the healthy population (5.4% in our cohort vs. 3.5% in healthy population) (13, 35). Interestingly, both PWS girls with CPP in our cohort had mUPD, but not all girls with mUPD have precocious puberty, suggesting that mUPD is not a predictor of CPP in patients with PWS.

We aimed to employ the newest normative data (“pubertograms”), thanks to which the age at onset of pubarche, gonadarche and further progression of puberty in our patients could be objectively evaluated. Most previous studies have concluded that puberty is delayed or incomplete in patients with PWS (36). However, our results have shown that the original concept of delayed puberty onset was modified by our results showing that in PWS patients the gonadarche occurs at a similar age to the healthy population consistently with others studies (36–38), occurring at a typical age of 10.0 years in girls and 11.7 in boys. On the other hand, pubarche occurs in both sexes substantially earlier than in the healthy population. Age at pubarche is correlated neither with age at GH treatment initiation, nor with BMI (39). However, BA in PWS patients was advanced by 0.7 years, and is significantly correlated with pubertal onset (gonadarche). In a previous study, accelerated BA was associated with GH treatment, which is known to accelerate skeletal maturation (40). This may also be causative in our patients. Alternatively, accelerated BA can be caused by the early onset of androgen production, premature pubarche (adrenarche) and obesity as observed by other observational association studies (41, 42), however the natural course of pubarche cannot be evaluated. Another factor influencing the early or late onset of puberty and reproductive physiology in PWS patients may be hyperleptinemia, which is derived from body composition and the number of adipocytes (43, 44).

We have confirmed that pubarche progression in adolescents with PWS is prolonged by over 1 year compared to the healthy population in both sexes. However, we failed to prove that there was no difference in breast development and testicular volume progression compared to the healthy population. The recent study (37) together with our paper, consistently show that these patients do not reach the adult stage of pubertal development without gonadal hormone replacement. Thus, hormone replacement is not needed to initiate puberty but is essential for its progression to later stages and for reaching the adult stage of maturation. Therefore, the early onset of pubarche and abnormal pubertal pattern is suggestive of a dissociation pattern from adrenal maturation leading up to adrenarche, and the functionality of the hypothalamic-pituitary-gonadal axis in these patients.

The relationship between genotype and phenotype in PWS patients is complex and is influenced by specific genetic mechanisms leading to the disorder (4). Understanding these genotype-phenotype correlations can help in managing and providing tailored care for individuals with PWS. However, we have shown that this is not applicable to pubarche, gonadarche, BA and pubertal progression. The onset and progression of puberty in PWS patients cannot be predicted by their genotype.

## Limitations

The clinical assessment of puberty was one of the most significant challenges. Therefore, we conducted a search for recent studies of pubertal development in populations of similar ethnicity and socioeconomic standard to allow comparison for our cohort with patients with PWS. For comparison, we used reference standards from Denmark (28–30). Despite this, due to the fact that it is not the same population, this limitation remains. If we eliminated this limitation, we would not be able to evaluate pubertal development in a standard way.

Another limitation was that only pubarche or only incipient signs of gonadarche were present in some of our patients. Therefore, it was not possible to evaluate the further progression of these signs in the whole cohort and part of these results were influenced by the size of the group. We will have more accurate information about the progression of puberty in these patients in a few years.

## Conclusion

Regardless of the genetic subtype and the *MKRN3* gene status, children with PWS had early and slowly progressing pubarche. However, the age at gonadarche was normal and further progression of puberty was insufficient. The BA at the onset of gonadarche was advanced and was significantly associated with gonadarche. Genetic background was not suitable predictor for the timing of pubertal onset.

## Data Availability

The raw data supporting the conclusions of this article will be made available by the authors, without undue reservation.
